# Contemporary radiooncological management of bone metastases from breast cancer: factors associated with prescription of different fractionation regimens (short or long course) in a rural part of North Norway with long travel distance

**DOI:** 10.1080/22423982.2016.1270080

**Published:** 2017-01-19

**Authors:** Carsten Nieder, Astrid Dalhaug, Ellinor Haukland, Bård Mannsåker, Adam Pawinski

**Affiliations:** ^a^ Department of Oncology and Palliative Medicine, Nordland Hospital, Bodø, Norway; ^b^ Department of Clinical Medicine, Faculty of Health Sciences, UiT- The Arctic University of Norway, Tromsø, Norway

**Keywords:** Breast cancer, bone metastases, prognostic factors, radiotherapy, palliative therapy

## Abstract

The aim of this study was to reduce barriers that prevent implementation of evidence-based recommendations about single-fraction palliative radiotherapy (PRT) and to demonstrate that single-fraction PRT yields similar outcomes as long-course treatment (≥10 fractions) in patients with bone metastases from breast cancer. This retrospective study (2007–2014) included 118 Norwegian female patients. All patients received guideline-conform systemic therapy including bone-targeting agents. Median survival was 12.7 months. Long-course PRT was prescribed in 60% of patients, while 21% had PRT with a single fraction of 8 Gy to at least one target. Reirradiation rate was not significantly higher after 8 Gy (9%, compared to 5% after long-course PRT and 6% after 4 Gy x5). Patients with favorable baseline characteristics such as younger age and good performance status (PS) were significantly more likely to receive long-course PRT. Biological subtype and comorbidity did not correlate with fractionation. Prognosis was influenced by biological subtype, extra-skeletal disease extent, severe anemia and abnormal CRP. The limited need for reirradiation after single fraction PRT might encourage physicians to prescribe this convenient regimen, which would improve resource utilization. Even patients with PS3 had a median survival of 3 months, which indicates that they could experience worthwhile clinical benefit.

## Introduction

Skeletal-related events (SRE) are common in patients with bone metastases from breast cancer [1–4]. The term SRE relates to pathological fractures, orthopaedic surgery, spinal cord compression and utilisation of radiotherapy. Jensen et al. estimated the incidence of bone metastases and SRE in newly diagnosed breast cancer patients in Denmark from 1999 to 2007 using the Danish National Patient Registry [5]. Of the 35,912 patients with breast cancer, 178 (0.5%) presented with bone metastases at the time of primary breast cancer diagnosis, and of these, 43% developed an SRE during follow-up. A total of 1,272 of 35,690 (3.6%) patients without bone metastases at diagnosis developed early bone metastases, i.e. during a median follow-up time of 3.4 years. Among these patients, 46% subsequently developed an SRE. The 5-year survival rate of Danish patients with bone metastases was 8% [6].

Radiotherapy for painful uncomplicated bone metastases is more common than radiotherapy for spinal cord compression or after orthopaedic stabilisation of fractures [4]. Multiple randomised studies suggest equivalent pain relief from bone metastases after radiotherapy with long-course regimens (≥10 fractions) and shorter courses [7–11]. Although American Society for Radiation Oncology evidence-based guidelines and the Choosing Wisely campaign endorse single-fraction treatments and caution against the use of extended courses, publications report modest single-fraction utilisation rates [12,13]. In the case of breast cancer with comparatively favourable survival rates, clinicians might overrate the importance of local control as a function of higher biologically equivalent dose in the era of effective systemic treatment. They might be reluctant to prescribe shorter courses because of the higher rates of reirradiation reported in previous studies [8] and a fear of skeletal complications from insufficiently controlled metastases. The purpose of the present retrospective quality of care audit is to compare patients who received long-course and short-course regimens, and to reduce barriers that prevent implementation of evidence-based recommendations. The latter is especially important in rural areas with large travel distance and challenging winter climate.

## Material and methods

### Patients and treatment

This retrospective intention-to-treat study included 118 consecutive female patients with bone metastases from breast cancer who received palliative external beam radiotherapy at the authors’ institution in Bodø (academic teaching hospital in rural North Norway). All patients were treated with linear accelerators between 2007 and 2014 after two- or three-dimensional treatment planning. Some patients presented with bone metastases at first cancer diagnosis, others later during the disease trajectory, and all had histological confirmation of malignancy. Systemic treatment was given according to the guidelines of the Norwegian Breast Cancer Group, which are stratified by biological subtype and available online. While single-fraction irradiation was recommended for uncomplicated bone metastases, final decision-making was left to the discretion of the treating physician. Nationwide, the proportion of single-fraction treatments has increased from 16% in 1997 to 41% in 2007 [14]. Radionuclide treatment was not utilised in this patient cohort.

### Blood tests

Serum lactate dehydrogenase (LDH), albumin, haemoglobin, C-reactive protein (CRP), calcium and alkaline phosphatase (ALP) were part of routine blood chemistry and imaging assessment in patients with metastatic breast cancer. However, some patients had missing values. The hospital’s electronic patient record system was used to collect all follow-up and baseline data including blood tests. The latter had to be no older than 2 weeks before the first fraction of radiotherapy. Elevated LDH was defined as ≥205 U/l according to the hospitals’ reference value (low albumin <34 g/l; high ALP ≥105 U/l; high calcium >2.55 mmol/l; normal CRP <5 mg/l; low haemoglobin <11.7 g/dl; blood transfusion was given if haemoglobin was <10 g/dl).

### Statistical methods

Actuarial survival from the first day of radiotherapy was calculated with the Kaplan–Meier method and compared between subgroups with differing baseline characteristics including, but not limited to, performance status, biological subtype and patterns of metastatic disease with the log-rank test. If the p-value was ≤0.05, the corresponding baseline characteristic was included in the multivariate analysis of prognostic factors for survival, which consisted of Cox regression (backward conditional method). Associations between different variables of interest were assessed with the chi-square or Fisher exact probability test (two-tailed). A p-value ≤0.05 was considered statistically significant.

## Results

### Patient characteristics

While 17 patients (15%) had bone metastases at initial diagnosis or *de novo* stage IV disease, the vast majority presented with metastatic disease at later time points. The median age of all patients was 61 years (range 33–87 years). The median time interval from the initial diagnosis of breast cancer was 57 months (range 1–384 months) and the median time interval from metastatic disease, irrespective of site, to palliative radiotherapy for bone metastases was 7 months (range 1–180 months). Further patient characteristics are shown in Table 1.

**Table 1.** Patient characteristics before radiotherapy (RT), n = 118.

Parameter	n	%
Triple negative^a^	22	20
ER positive HER2 negative^a^	68	62
ER negative HER2 positive^a^	5	5
ER and HER2 positive^a^	15	14
Metastases at first diagnosis^a^	17	15
Metachronous metastatic disease^a^	98	85
Interval from met. disease to RT ≤6 mo^a^	56	49
Interval from met. disease to RT >6 mo^a^	59	51
No extra-skeletal visceral metastases	57	48
Visceral metastases	61	52
Liver metastases	36	31
No liver metastases	82	69
Lung metastases	38	32
No lung metastases	80	68
Pleural metastases/effusion	19	16
No pleural metastases/effusion	98	84
Metastatic spinal cord compression	10	8
No metastatic spinal cord compression	108	92
ECOG PS 0	32	27
ECOG PS 1	38	32
ECOG PS 2	32	27
ECOG PS 3–4	16	14
Charlson comorbidity index 0^a^	58	52
Charlson comorbidity index ≥1^a^	53	48
Age <65 years	70	59
Age ≥65 years	48	41
Spinal target volume	50	42
No spinal target volume	68	58
Only one target volume	60	51
More than one target volume	58	49
Reirradiation	18	15
No reirradiation	100	85
Serum albumin normal^a^	94	84
Serum albumin low^a^	18	16
Serum LDH normal^a^	45	45
Serum LDH high^a^	56	55
Serum ALP normal^a^	47	45
Serum ALP high^a^	58	55
Serum CRP normal^a^	55	50
Serum CRP high^a^	56	50
Haemoglobin normal^a^	80	71
Haemoglobin low^a^	33	29
Received blood transfusion before RT	9	8
No blood transfusion	109	92
Hypercalcemia^a^	10	8
No hypercalcemia^a^	101	91
Non-opioid analgesics^a^	74	69
Opioid analgesics^a^	33	31
Steroids^a^	31	30
No steroids^a^	72	70

a information not available in all patients

ER: oestrogen receptor; ECOG PS: Eastern Cooperative Oncology Group performance status; LDH: lactate dehydrogenase; ALP: alkaline phosphatase; CRP: C-reactive protein

### Treatment details

Few patients (5%) received more than 10 fractions. The most common fractionation regimen was 10 fractions of 3 Gy (55%) followed by 5 fractions of 4 Gy (24%) and 8 Gy single fraction (16%). The proportion of patients treated with long-course radiotherapy remained unchanged over time. Fifty patients (42%) had spinal target volumes, but only 8% had a diagnosis of metastatic spinal cord compression (MSCC). Forty-two patients (36%) received simultaneous radiotherapy to two target volumes and 16 (14%) to at least three target volumes. The vast majority of patients with more than one target volume received the same fractionation regime for all volumes. However, 8 patients with differing regimes had both long and short-course radiotherapy during the same treatment course. Therefore, 25 patients (21%) had at least one target volume that was treated with a single fraction of 8 Gy. Eighteen patients (15%) entering the study received reirradiation to a previously treated skeletal target volume. All but one patient completed their prescribed course of radiotherapy.

### Factors associated with prescription of long-course radiotherapy

As shown in Table 2, significantly more patients with favourable baseline characteristics received long-course regimens. These characteristics included absence of lung metastases (hazard ratio (HR) with 95% confidence interval 1.85 (1.21–2.83)) and/or pleural metastases/effusion (HR 1.82 (1.18–2.82)), normal serum haemoglobin (HR 2.43 (1.43–4.15)), CRP (HR 1.64 (1.20–2.26)), LDH (HR 1.64 (1.22–2.21)) and albumin (HR 2.41 (1.13–5.15)), i.e. surrogate markers of disease extent, early radiotherapy within 6 months from diagnosis of metastatic disease (HR 1.71 (1.19–2.45)), age younger than 65 years (HR 1.43 (1.02–2.00)), and good performance status (Eastern Cooperative Oncology Group (ECOG) performance status 0–1) (HR 1.75 (1.22–2.52)). Biological subtype (HER2 and oestrogen receptor status), comorbidity and treatment of spinal/multiple target volumes were not associated with fractionation regimen.

**Table 2.** Choice of fractionation stratified by baseline characteristics (n = 118).

Parameter	n	% <10 fractions (ITT)	% ≥10 fractions (ITT)	p-value
Triple negative^a^	22	36%	64%	
ER positive HER2 negative^a^	68	35%	65%	
ER negative HER2 positive^a^	5	20%	80%	
ER and HER2 positive^a^	15	53%	47%	0.61^b^
Metastases at first diagnosis^a^	17	35%	65%	
Metachronous metastatic disease^a^	98	41%	59%	0.84
Interval from met. disease to RT ≤6 mo^a^	56	27%	73%	
Interval from met. disease to RT >6 mo^a^	59	53%	47%	**0.007**
No extra-skeletal visceral metastases	57	33%	67%	
Visceral metastases	61	46%	54%	0.28
Liver metastases	36	44%	56%	
No liver metastases	82	38%	62%	0.76
Lung metastases	38	58%	42%	
No lung metastases	80	31%	69%	**0.017**
Pleural metastases/effusion	19	64%	36%	
No pleural metastases/effusion	98	35%	65%	**0.038**
Metastatic spinal cord compression	10	20%	80%	
No metastatic spinal cord compression	108	42%	58%	0.27
ECOG PS 0	32	28%	72%	
ECOG PS 1	38	26%	74%	
ECOG PS 2	32	50%	50%	
ECOG PS 3–4	16	75%	25%	**0.01^b^**
Charlson comorbidity index 0^a^	58	34%	66%	
Charlson comorbidity index ≥1^a^	53	42%	58%	0.46
Age <65 years	70	31%	69%	
Age ≥65 years	48	52%	48%	**0.01**
Spinal target volume	50	36%	64%	
No spinal target volume	68	43%	57%	0.45
Only one target volume	60	35%	65%	
More than one target volume	58	45%	55%	0.50
Reirradiation	18	61%	39%	
No reirradiation	100	36%	64%	0.07
Serum albumin normal^a^	94	33%	67%	
Serum albumin low^a^	18	72%	28%	**0.007**
Serum LDH normal^a^	45	18%	82%	
Serum LDH high^a^	56	50%	50%	**0.003**
Serum ALP normal^a^	47	30%	70%	
Serum ALP high^a^	58	43%	57%	0.21
Serum CRP normal^a^	55	24%	76%	
Serum CRP high^a^	56	54%	46%	**0.002**
Haemoglobin normal^a^	80	26%	74%	
Haemoglobin low^a^	33	70%	30%	**0.0001**
Received blood transfusion before RT	9	67%	33%	
No blood transfusion	109	38%	62%	0.22
Hypercalcemia^a^	10	30%	70%	
No hypercalcemia^a^	101	40%	60%	0.36
Non-opioid analgesics^a^	74	50%	50%	
Opioid analgesics^a^	33	27%	73%	0.06
Steroids^a^	31	39%	61%	
No steroids^a^	72	42%	58%	0.81

a information not available in all patients

b p-value calculated over all strata

ER: oestrogen receptor; ITT: intention to treat; ECOG PS: Eastern Cooperative Oncology Group performance status; LDH: lactate dehydrogenase; ALP: alkaline phosphatase; CRP: C-reactive protein

### Reirradiation of target volumes irradiated for the first time in the context of this study

Since 18 out of all 118 patients included in this study received reirradiation prior to entering the study, we excluded these patients from the analysis of reirradiation during follow-up. The remaining 100 patients received radiotherapy to a total of 154 target volumes. The proportions of reirradiated patients were low and not significantly associated with fractionation regimen (9% after single-fraction PRT, 5% after long-course PRT and 6% after 4 Gy ×5, p=0.4 and 1.0, respectively). There was no need for orthopaedic surgery in irradiated regions during follow-up.

### Overall survival

**Figure 1.**
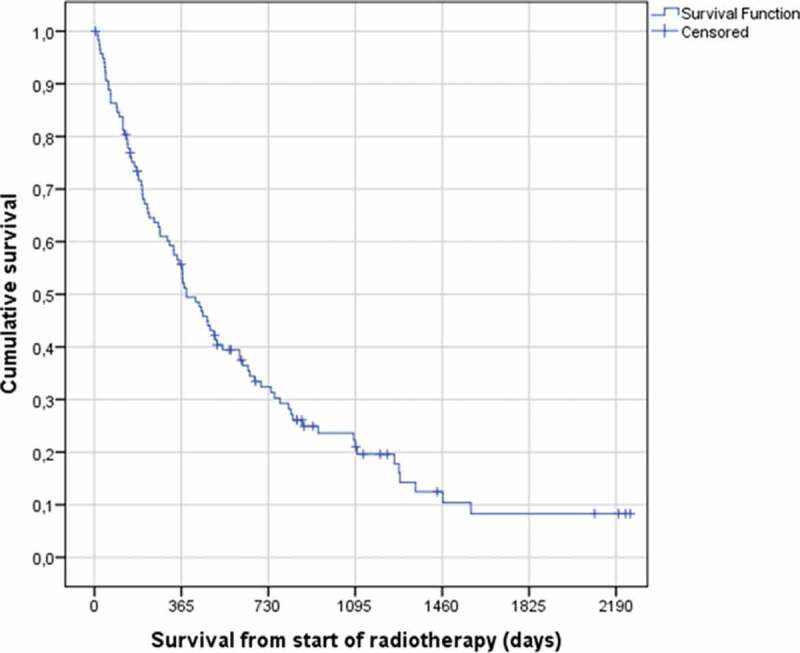
Actuarial Kaplan–Meier survival curve for 118 patients with bone metastases from breast cancer (median 12.7 months). Nearly 10% of the patients were still alive after 6 years.

Twenty-five patients were alive at last follow-up (15 June 2015) with a median follow-up of 28 months. Date of death was known in all other patients. Median survival was 12.7 months and 22% of the patients were alive after 3 years (Figure 1). Five patients (4%) received radiotherapy in the last 30 days of life and 13 (11%) in the last 2 months. Median survival from initial diagnosis of metastatic disease was 20 months. Of all parameters shown in Table 1 or reported in the results section, 6 were found to be independent prognostic factors for survival after radiotherapy in multivariate Cox regression analysis: absence of extra-skeletal metastases (p=0.0001), no pleural metastases/effusion (p=0.0001), CRP <5 mg/l (p=0.0001), positive oestrogen receptor (p=0.001), no lung metastases (p=0.001), no need for blood transfusion because of anaemia (p=0.048). Radiotherapy-related parameters such as fractionation were not statistically significant. Neither was performance status. Even patients with ECOG performance status 3 had a median survival of 3 months.

## Discussion

We performed a comprehensive analysis of fractionation patterns and clinical endpoints including reirradiation rate and survival after radiotherapy for bone metastases from breast cancer. In our country’s publicly funded healthcare system all patients have equal access to treatment with full reimbursement of travel and housing expenses. Even in our rural area, the utilisation of palliative radiotherapy did not vary with distance between residence and hospital [15]. Approximately 60,000 inhabitants live within 60 km of the radiotherapy facility and another 150,000 in remote areas (maximum 400 km) including small islands. Copayments for chemo- or radiotherapy do not exist. Adherence to national guidelines is excellent. Compared with other countries, the proportion of single-fraction treatments for bone metastases, 41% in 2007 for all tumour types combined [14], was quite high. However, there were substantial differences in the proportion of single-fraction treatments between the treatment centres (range 25–54%). These differences persisted after adjustment for sex, age, primary diagnosis, anatomical region, and travel distance. Because of limited nationwide capacity and lower than recommended general utilisation of radiotherapy, waiting lists existed in all radiotherapy departments despite operating at maximum capacity. Because there is no reason to believe that patient characteristics differed to the same degree as single-fraction utilisation rates, factors such as education and local traditions might have played a role. Undoubtedly, oncologists have to make individual decisions, which may be influenced by different criteria. Ideally, they would rely on objectively measurable validated parameters, which are related to the outcome of interest. In the context of this study, we assumed that long-course radiotherapy (≥10 fractions) should be reserved for a minority of patients with bone metastases from breast cancer, e.g. those with MSCC. We examined factors associated with prescription of such regimens among patients in our centre.

A typical patient in our study was a postmenopausal woman with oestrogen receptor-positive and HER2-negative disease who developed metachronous uncomplicated bone metastases and continued on state-of-the-art systemic therapy. According to the national guidelines, such patients receive zoledronic acid and several lines of sequential hormonal and cytotoxic therapy, depending on visceral tumour load, response to previous therapy and other parameters. We found that surprisingly many patients (60%) were treated with long-course radiotherapy, and that these figures had remained constant over time. Reirradiation rate was numerically higher after single-fraction radiotherapy (9%, compared with 5% after long-course therapy and 6% after 5 fractions of 4 Gy), but these differences were not statistically significant. Previous studies that included patients with different primary tumours reported reirradiation in 11–42% after a single fraction and 0–24% after multiple fractions [8]. In our study, orthopaedic surgery was not required in any patient. No survival difference was evident either. These findings suggest that short-course radiotherapy is oncologically safe in the current era of effective systemic therapy.

Limitations of this study include the number of patients, statistical power of subgroup analyses, and retrospective design. In a larger cohort of patients, the different reirradiation rates might have reached the level of statistical significance. Nevertheless, this would not imply clinical relevance. Patients with favourable baseline characteristics were significantly more likely to receive long-course regimens. These characteristics included absence of lung metastases and/or pleural metastases/effusion, normal serum haemoglobin, CRP, LDH and albumin (surrogate markers of disease extent), early radiotherapy within 6 months from diagnosis of metastatic disease, age younger than 65 years, and good performance status (ECOG 0–1). Biological subtype (HER2 and oestrogen receptor status), comorbidity and treatment of spinal/multiple target volumes did not correlate with fractionation. A possible interpretation is that clinicians are afraid of providing inadequate treatment to the young, recently diagnosed and well-functioning patients. It is necessary to confirm these results in larger studies. There is currently no sufficient evidence to suggest that local treatment improves the survival time of patients with oligometastatic bone metastases who receive effective systemic therapy. In order to change the observed pattern of care, physicians need to be aware of the excellent results obtained with short-course radiotherapy. Such treatment is also adequate in elderly patients and those with limited performance status, given that even patients with PS3 had a median survival of 3 months, which indicates that they could experience worthwhile clinical benefit after PRT. In other words, survival expectation in these subgroups is sufficiently long to warrant optimal pain control. Patients responding to radiotherapy commonly do so within approximately 4 weeks [16,17].

Disease extent in patients with metastatic breast cancer is highly variable, ranging from solitary bone metastasis to widespread bone marrow involvement, and often including extra-skeletal sides such as lung, pleura, liver and lymph nodes [18]. Thus, survival of these patients might vary from few months to several years. We found a large number of patient and disease characteristics to be associated with survival after radiotherapy. These included, for example, breast cancer type (shortest survival for triple negative status, median 5.5 months) and patterns of metastatic disease (longest survival for bone-only disease, median 22.9 months), but not age. Interestingly, performance status was less important than other factors. A potential explanation might be that poor performance status resulting from bone pain often improves rapidly after radiotherapy.

Gebhardt et al. reported an effective method to improve decision-making [19]. Their group implemented a clinical pathway for the management of bone metastases in 2003 that required the entry of management decisions into an online tool that subjected off-pathway choices to peer review beginning in 2009. In 2014, the pathway was modified to encourage single-fraction treatment and the use of >10 fractions was considered off pathway. They evaluated data from 16 integrated sites from 2003 through 2014. Overall, 12,678 unique courses were delivered. From 2003 to 2008, the single-fraction utilisation rate was 8%. This increased to 11% from 2009 to 2013 and to 16% in 2014. Use of >10-fraction regimens significantly decreased from 19% in 2003–2008 to 10% in 2014. By 2014, >90% of courses were delivered with <10 fractions. Comparable efforts were undertaken regarding hypofractioned radiotherapy for non-metastatic breast cancer [20]. These studies demonstrate that provider-driven clinical pathways are able to standardise practice patterns and promote change.

## Conclusions

The likelihood of receiving long-course radiotherapy was significantly higher in younger patients, those with good performance status, limited disease extent, and shorter time interval since diagnosis of metastatic disease. The limited need for reirradiation after single-fraction PRT should encourage physicians to consider this convenient regimen, which is also suitable for patients with reduced performance status and has been shown in the literature to improve quality of life across all subgroups. Single-fraction PRT contributes to optimal resource utilisation and improves access to treatment, especially for frail patients and those with larger travel distance. Decision-making should take into account that patients from remote regions need to spend variable amounts of time away from their relatives and friends. This fact becomes increasingly important in the terminal stage of disease, when the remaining lifetime is short and active treatment unlikely to extend survival.

## Declarations

**Ethics approval**: As a retrospective quality of care analysis, no approval from the Regional Committee for Medical and Health Research Ethics (REK Nord) was necessary (national policy in Norway). This research project was carried out according to our institutions’ guidelines and with permission to access the patients’ data.

**Consent for publication**: Not applicable.

**Availability of data and materials**: Data will not be shared, but a copy of relevant baseline parameters can be provided to researchers attempting to pool data from several institutions for large-scale analyses.
